# Digital Intervention in Children With Developmental Language Disorder: Systematic Review

**DOI:** 10.2196/59992

**Published:** 2025-05-23

**Authors:** Zhaowen Zhou, Cheng Deng, Dongling Yin, Qiaoxue Yang, Zhuoming Chen

**Affiliations:** 1 Department of Rehabilitation Medicine, The First Affiliated Hospital of Jinan University, 613 Huangpu Avenue West, Guangzhou, 510630, China, 86 13392692183; 2 Department of Children’s Health Care, Zhongshan Torch Development Zone People’s Hospital, Zhongshan, China

**Keywords:** developmental language disorder, digital intervention, systematic review, language rehabilitation, phonological skills

## Abstract

**Background:**

Developmental language disorder (DLD) is one of the most common neurodevelopmental disorders. Effective intervention is primarily important for improving the language and communication skills of children with DLD, and strengthening these skills ensures quality of life and prevents negative effects in adulthood. Digital interventions have the potential to complement conventional language intervention, reducing the workload for therapists and increasing accessibility to language training in homes or schools.

**Objective:**

This systematic review aimed to explore the language domain that is most frequently targeted by digital intervention in children with DLD.

**Methods:**

The study protocol was registered in the International Prospective Register for Systematic Reviews (PROSPERO) and was ascribed the CRD42023477946 registration code. The initial search was conducted on May 2023 from 4 databases: “PubMed,” “Scopus,” “PsycInfo,” and “IEEE Xplore,” following a method adapted from PRISMA (Preferred Reporting Items for Systematic Reviews and Meta-Analyses). Inclusion criteria include studies recruiting patients diagnosed with DLD; studies that reported digital interventions based on apps, video games, augmented reality, or any other type of software based on language outcomes; and English language studies. Reviews, letters, conference proceedings, abstracts, editorials, and studies not published in English were removed. The titles and abstracts of the identified records were initially screened and selected by 2 independent and blinded reviewers. Data extraction and quality assessment were performed by 3 independent reviewers.

**Results:**

Overall, 13 studies were included; 961 children with DLD underwent a digital intervention. The mean age ranged from 3.47 (SD 0.17) to 11.19 (SD 1.12) years. A total of 8 were randomized controlled trials, and 5 were quasi-experimental studies. Targeting domains of digital intervention were phonological skills (n=5), general language function (n=3), grammar (n=3), and vocabulary (n=2).

**Conclusions:**

This systematic review indicates that phonological skills are the most frequently targeted language domain by digital interventions in children with DLD. Given the limited number and the heterogeneity of the studies included, it is still unclear whether digital intervention was effective in improving different language skills in children with DLD. There was less evidence supporting its effectiveness in expressive language skills, which indicates a need to update expressive language digital training programs in the future. Further higher-level evidence, such as randomized controlled trial studies in this area, is needed to direct the development of digital programs.

## Introduction

Developmental language disorder (DLD) is one of the most prevalent neurodevelopmental disorders that has profound and lasting effects on individual development [1]. It occurs in approximately 7.6%-8.5% of preschool children around the world [2,3]. According to the *International Classification of Diseases, Eleventh Revision*, DLD is characterized by persistent deficits in the acquisition, understanding, production, or use of language (spoken or signed), that arise during the developmental period, typically during early childhood, and cause significant limitations in the individual’s ability to communicate. The individual’s ability to understand, produce, or use language is markedly below what would be expected given the individual’s age. Language deficits are not explained by another neurodevelopmental disorder, or sensory impairment, or neurological condition, including the effects of brain injury or infection [4]. The term DLD has been suggested to replace earlier terms, such as specific language impairment, language impairment, language disorder, and primary spoken language disorder. However, for clarity purposes, in this review, we will use DLD, although due to its recent introduction, the studies included in this work used specific language impairment as a diagnostic label.

Language development is a critical domain of children’s overall development. Language skills are an important means of communication, which comprises the ability to send and receive information through oral and written language [5]. DLD can affect different aspects of language processing, such as the form of language (phonetic, phonological, morphological, morpho-syntactic, and syntactic processing); content (semantic-lexical and phrasal processing); and use (pragmatic and discursive processing) [6]. Individuals with DLD may not only experience difficulties in communication but also face challenges from other different domains, such as reduced engagement in playing and academic learning [7]. DLD may also increase the risk of poor health-related quality of life [8], including mental health problems such as anxiety and depression [9,10]. In addition, DLD has an impact on the development of cognitive functioning [11,12], sensorimotor functioning [11,13], or behavioral functioning [14]. DLD can coexist with many other conditions, such as learning disorders, delayed motor milestones, auditory processing disorders, attention-deficit/hyperactivity disorder, lack of coordination, and other motor disorders [15,16]. The impact of DLD may persist to adulthood and have long-term effects on cognitive function, community function, interpersonal relationships, and employment [17-19]. Therefore, effective interventions during childhood are essential to improve the quality of life of individuals with DLD and to prevent further negative effects in adulthood.

Convention interventions for DLD are usually conducted one by one in person by speech and language pathologists (SLPs). During treatment sessions, SLPs will design challenging interventions for children using interesting toys and activities to stimulate children’s language skills and teach language learning strategies. However, that requires SLPs to be well-trained and experienced. Limited by the number of therapists, the geographic distance between the SLP and the recipient of services, and economic conditions, in-person training is not always available to children with DLD [20].

Digital interventions have the potential to be used as an adjunct to conventional language intervention, which could reduce therapists’ workload and increase children’s accessibility to language training in settings such as homes or schools, enabling them to practice under the supervision of caretakers or teachers [21]. Digital intervention provides repeated training of particular skills easier; it motivates children by giving automated timely feedback in a game-like format [22]; digital intervention can also be programmed to respond adaptively to the child’s level of performance so that training is focused on materials that are just beyond current competence; every response made by the child can be detailly recorded for further analysis. In the economic aspect, digital intervention also has the potential to increase accessibility to care, reduce patients’ travel and costs, and develop culturally appropriate services, especially for different language speakers [23].

There is a wide range of digital interventions for children with DLD that are aimed at different language domains. This review aimed to systematically analyze which language domains are most frequently supported by digital interventions for children with DLD.

## Methods

### Study Identification

This systematic literature review was performed according to the methodology described in the Cochrane Handbook for Systematic Reviews [24] and was reported based on the PRISMA (Preferred Reporting Items for Systematic Reviews and Meta-Analyses) statement for reporting systematic reviews [25]. Additionally, the study protocol was registered in the International Prospective Register for Systematic Reviews (PROSPERO) and was ascribed to the CRD42023477946 registration code.

The initial search was conducted on May 2023 by searching the databases “PubMed,” “Scopus,” “PsycInfo,” and “IEEE Xplore” using the following search terms: ((“developmental language disorder” OR “language delay” OR “speech delay” OR “language impairment*” OR “speech impairment*” OR “language disorder*” OR “speech disorder*” OR “language difficult*” OR “speech difficult*”) AND (“Child*” OR “Preschool”) AND (“computer-based” OR “computer assisted therapy” OR “computer games” OR “software” OR “websites” OR “computer*” OR “digital*” OR “electronic” OR “gaming” OR “internet*” OR “video game*” OR “online” OR “on-line” OR “web*”)).

No limitations in the search strategy were applied to the publication date, study design, or language. References of considered studies were also searched to identify any further relevant data.

The records identified by the search were uploaded to “Rayyan” [26] to organize the study selection more efficiently. The titles and abstracts of the identified records were initially screened and selected by 2 independent and blinded reviewers (ZZ and CD) based on their pertinence to the review topic. Conflicts and disagreements were resolved by consensus.

The following set of predefined inclusion criteria were then individually applied to the selected studies in their full-text version: (1) studies recruiting patients diagnosed with DLD; (2) studies that reported digital interventions based on apps, video games, augmented reality, or any other type of software based on language outcomes; and (3) English language studies.

Reviews, letters, conference proceedings, abstracts, editorials, case reports, and case series were excluded. Studies not published in English were removed. Studies in which the targeted populations were children with cognitive delay, deafness, autism spectrum disorders, genetic syndromes (Down syndrome and Klinefelter syndrome), neurological deficits, pervasive developmental disorders, traumatic brain injuries, primary disorders (sensory, neurological, and psychiatric), children with dysphonia, dysarthria, dysrhythmias or stuttering, dyslalia or specific speech articulation disorder, and bilingualism were excluded.

### Data Extraction and Quality Assessment

Data extraction and quality assessment were performed by 3 independent reviewers (ZZ, CD, and DY). The extracted data and results of the quality assessment are reported in Multimedia Appendix 1 [22,27-38]. The quality of retrieved studies was assessed by the Joanna Briggs Institute (JBI) appraisal tool [39]. The JBI checklist for randomized controlled trials (RCTs) [40] and for quasi-experimental studies [41] was used according to the study design of included studies. JBI appraisal tool evaluates internal validity and statistical conclusion validity of the targeted studies. For RCTs, questions related to internal validity were further separated to assess bias related to selection and allocation, administration of intervention or exposure, assessment, detection and measurement of outcome, and participant retention, and for quasi-experimental studies, domains of internal validity include the bias related to temporal precedence, selection and allocation, confounding factors, administration of intervention or exposure, assessment, detection, and measurement of the outcome, and participant retention.

The purpose of this appraisal is to assess the methodological quality of studies and to determine the extent to which a study has addressed the possibility of bias in its design, implementation, and analysis. Studies with percentage scores of <49% were classified as weak, studies between 50% and 79% were moderate, and studies ≥80% were classified as high in quality with reduced bias. This classification has been used in prior systematic reviews [42].

### Treatment Fidelity Assessment

A 40-item checklist with definitions of intervention fidelity domains and relevant examples was used to assess the treatment fidelity [43]. The authors used the checklist to assess the presence of strategies identified in the 5 specific domains: treatment design, training providers, delivery, receipt, and enactment. Intervention fidelity information was categorized and coded as “present (1),” “not present but should be present (0),” or “not applicable (NA).” Authors coded studies independently and discussed and reconciled any discrepancies.

## Results

### Overview

**Figure 1.**
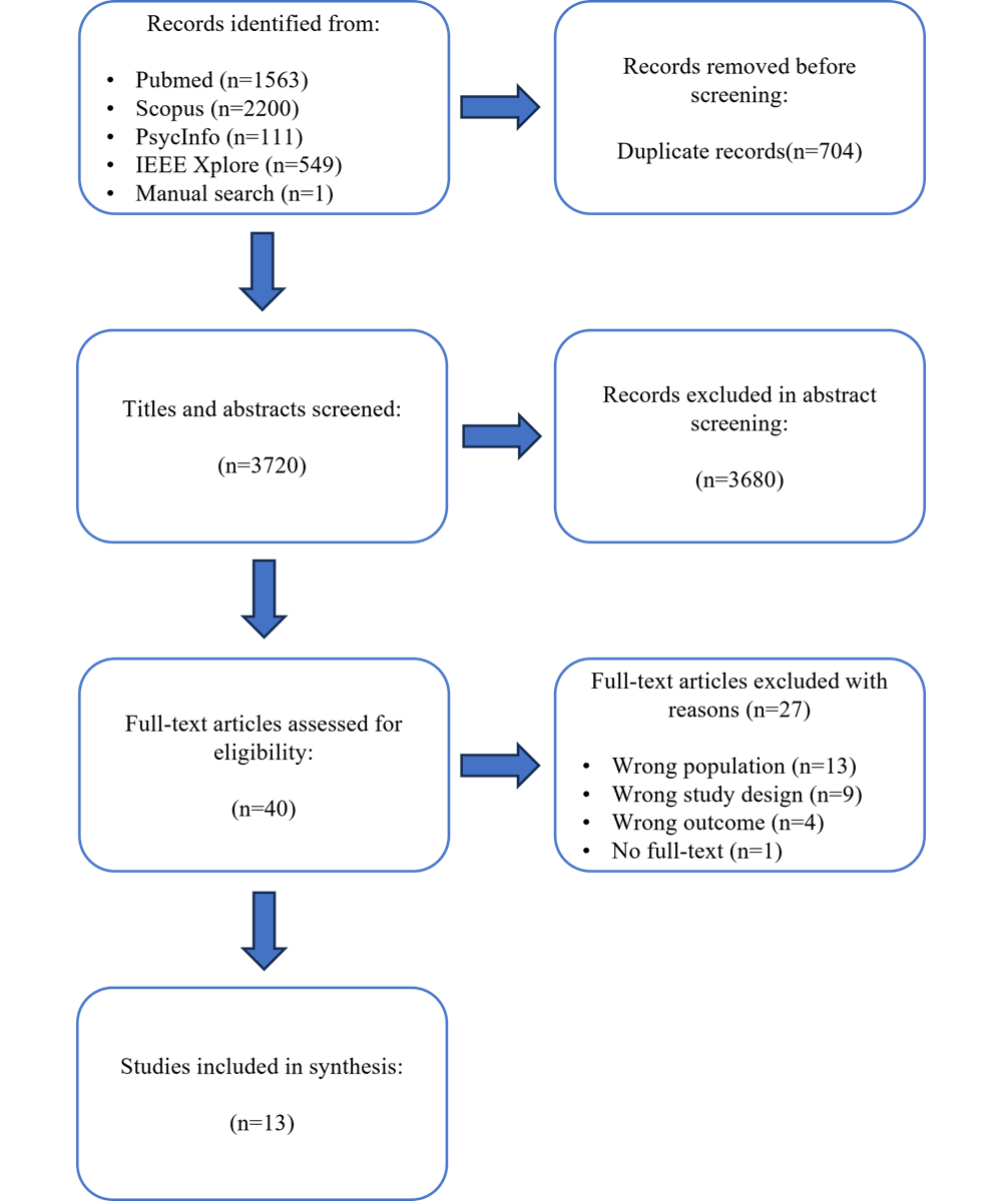
PRISMA flowchart describing the inclusion process of the studies. PRISMA: Preferred Reporting Items for Systematic Reviews and Meta-Analyses.

The following records were identified via electronic databases: PubMed (n=1563); Scopus (n=2200), PsycInfo (n=111), and IEEE Xplore (n=549), which yielded 4423 unique records. One additional record was identified via manual searching. The flow diagram in Figure 1 shows the records remaining at each stage and the reasons for exclusion of studies reviewed at the full-text stage. One full-text study could not be sourced for review and was excluded at the full-text stage. A total of 13 studies met the inclusion criteria for this study.

Of the 13 studies that met the inclusion criteria, 8 studies were RCTs [22,28-30,33-35,37], and 5 studies were quasi-experimental studies [27,31,32,36,38]. According to the quality assessment, 6 studies were of high quality, and 7 studies were of moderate quality out of the 13 studies. The oldest study was conducted in 2005, and the most recent study was conducted in 2022.

Overall, 961 children diagnosed with DLD were included, 63.89% (n=460) were male and 36.11% (n=260) were female (gender proportion not reported in 5 studies). Mean age ranges from 3.47 (SD 0.17) to 11.19 (SD 1.12) years old.

The digital intervention programs used included: Fast ForWord (FFW; n=6) [27], Reading Doctor (n=1) [28], 作業TipOn (n=1) [29], My Sentence Builder (n=1) [37], Jingyun Rehab Platform (n=1) [38], My PlayHome (n=1) [22], Dr. Neuronowski (n=1) [30], and a program developed by Heikkilä et al [31] and was run with Presentation software (Neurobehavioral systems; n=1). Among the 8 programs mentioned above, 7 of them are software for computers, tablets, or mobile phones, and Jingyun Rehab Platform is a website for computers, tablets, and mobile phones. A total of 4 of the programs were developed in English, 2 of them were developed in Chinese (作業TipOn and Jingyun Rehab Platform), 1 was in Finnish, and 1 was in Polish.

Of the 13 studies included, 5 studies had a follow-up of 5 weeks to 6 months after the end of the intervention. A total of 4 of these studies showed that the intervention effects of digital intervention on language functioning in children with DLD were maintained or improved at follow-up.

Phonological skills were the most frequently targeted skill in digital intervention for DLD (5 out of 13). General language function and grammar were each reported in 3 studies. Vocabulary was reported in 2 studies.

### Phonological Skills (n=5)

The effectiveness of digital intervention in phonological skills for children with DLD was reported in 5 studies. These studies were targeted at phonological skills, including phoneme awareness, letter-sound aptitude, and early decoding ability. The programs used to conduct phonological skills training in 5 studies were varied, including the Reading Doctor iPad app, 作業TipOn (mobile app), FFW*,* and Dr. Neuronowski. The duration of the digital intervention was 4 to 9 weeks. The main design of the game to train receptive phonological skills was based on improving basic auditory processing skills, either through nonspeech stimuli such as sounds and tones or through speech stimuli such as syllables and words. In these games, the sound or an audiovisual video of the targeted letter, syllables, or words was provided, and children were required to point to the best-matched picture among the distractors or type the correct spelling of the word. Significant improvement in receptive phonological skills was found in 4 studies [28-31] compared with the control group. Results showed that audiovisual speech might be more effective than auditory speech in training phonological skills in children. However, in the study targeted spelling [27], which is a higher level of phonological skills, trained groups did not differ from the untrained control group in spelling. In the studies targeting phonological skills, researchers also aimed to improve participants’ word learning and reading [27-29] through phonemic awareness training. Bishop et al [27] suggested that digital intervention did not improve word and nonword reading compared to the untrained group, whereas Carson [28] found a significant improvement in word reading skills in the digital intervention group compared to the control group. In addition, Chen and Lin [29] found that the total number of games played and the accuracy in games were significantly correlated with the performance of the prepost gain in the word definition task, which asked children to say the meaning of a vocabulary word they heard.

### General (n=3)

There were 3 studies [32-34] that examined interventions targeting more than one domain, including phonological skills, vocabulary, and grammar. The same digital program and intervention procedures were applied to both studies. Children participated in the FFW program for 1 hour and 40 minutes (100 min: five games of 20 min each) each weekday for 6 weeks (30 d). The intervention ended when they reached the dismissal criterion established by Scientific Learning Corporation of 90% completion on 5 of 7 exercises or until they exhibited plateaus in performance for 10 days before the 6-week end date. FFW included games that targeted discrimination of tones (viz, Circus Sequence), detection of individual phoneme changes (viz, Old McDonald’s Flying Farm), matching phonemes to a target (viz, Phoneme Identification), identifying matched syllable pairs (viz, Phonic Match), discriminating between minimal pair words (viz, Phonic Words), recalling commands (viz, Block Commander), and comprehending grammatical morphemes and complex sentence structures (viz, Language Comprehension Builder). RCTs conducted by Cohen et al [33] and Gillam et al [34] revealed that though participants gained improvement from FFW, there was no additional effect for computer intervention compared to conventional intervention. However, Loeb et al [32] found no significant improvement in phonological skills and reading.

### Grammar (n=3)

A total of 3 studies included grammar as the primary focus of digital intervention for DLD, of which, 2 studies examined the effectiveness of FFW, and 1 study investigated My Sentence. Both Hsu and Bishop [35] and Bishop et al [36] used FFW to implement receptive training of grammatical skills. Children heard a spoken sentence and then moved or activated objects on the computer screen to match the spoken sentence. Children in these two studies both showed greater accuracy in the training program, but showed no significant improvement on standardized tests of receptive grammar. Washington et al [37] used My Sentence to address expressive grammar deficits in DLD. Results showed that computer-based training significantly outperformed conventional intervention, but no significant differences in treatment gains were found between computer-based training and noncomputer-based training.

### Vocabulary (n=2)

Among the 13 studies included, 2 studies targeted vocabulary learning. Yi et al [38] and Zwitserlood et al [22] focus on the receptive vocabulary of children with DLD, using pictures of web-based platforms or software games. During the vocabulary training, Zwitserlood et al [22] designed play activities with the digital game, and exposed children to the targeted words with symbolic play sessions, narrative, natural interaction, and language facilitation strategies. Yi et al [38] presented the sound of words or syllables and pictures to the children and instructed them to point at the picture that matched the word best. The intervention periods of intervention for vocabulary skills were approximately 1-3 months. Both studies showed that digital interventions were beneficial for children’s vocabulary skills.

### Fidelity of the Studies

Table 1 presents the treatment fidelity for the included studies, and Multimedia Appendix 2 [22,27-38] presents the scores of fidelity for each study.

There are 2 domains in which the treatment fidelity scores were above 50%, including the “Treatment design” domain at 75% (SD 0.15%) and the “Enactment” domain at 58% (SD 0.18%). The lowest mean proportion of adherence to strategies was found in the “Receipt” domain, where, on average, only 4% (SD 0.09%) of strategies were reported among the studies. Finally, the mean proportion of adherence to strategies in the “Training providers” and “Delivery” domains was 16% (SD 0.17%) and 19% (SD 0.15%), respectively. The mean proportion of adherence to treatment fidelity strategies included across all 5 domains for all studies was 43% (SD 0.10%).

Based on the fidelity scoring by Borrelli [43], where 50% constitutes low-fidelity scoring, 69% (9/13) of the interventions scored a low treatment fidelity across all 5 domains. In total, 31% (4/13) of the studies, including Chen and Lin [29], Loeb et al [32], Gillam et al [34], and Zwitserlood et al [22], scored >0.50 in the overall treatment fidelity (range 0.50-0.58).

**Table 1.** Treatment fidelity for the included studies.

Fidelity domains	Treatment design	Training providers	Delivery	Receipt	Enactment	Overall fidelity proportion per study
Bishop et al [27]	0.63	0.14	0.11	0.00	0.50	0.34
Carson [28]	0.63	0.00	0.00	0.25	1.00	0.34
Chen and Lin [29]	0.88	0.14	0.44	0.00	0.50	0.53
Dacewicz et al [30]	0.88	0.14	0.22	0.00	0.50	0.47
Heikkilä et al [31]	0.88	0.29	0.00	0.00	0.50	0.45
Loeb et al [32]	0.88	0.29	0.22	0.00	0.50	0.50
Cohen et al [33]	0.88	0.00	0.33	0.00	0.50	0.47
Gillam et al [34]	0.88	0.29	0.33	0.00	0.50	0.53
Hsu and Bishop [35]	0.56	0.00	0.00	0.00	0.50	0.26
Bishop et al [36]	0.56	0.00	0.00	0.25	1.00	0.32
Washington et al [37]	0.81	0.29	0.11	0.00	0.50	0.45
Yi et al [38]	0.50	0.00	0.33	0.00	0.50	0.32
Zwitserlood et al [22]	0.88	0.57	0.33	0.00	0.50	0.58
Proportion, mean (SD)	0.75 (0.15)	0.16 (0.17)	0.19 (0.15)	0.04 (0.09)	0.58 (0.18)	0.43 (0.10)

## Discussion

### Principal Findings

This systematic review aimed to explore the language domain that is most frequently targeted by digital intervention in children with DLD. Phonological skills were the most researched skills among all included studies, especially receptive phonological skills. Phonological awareness is the skill of being aware of sounds in spoken language. It ranges from dividing words into syllables, recognizing and producing rhymes, and matching words with the same beginning sound, to more complex abilities such as separating and processing individual sounds or phonemes, which are causally related to early word decoding skills [44]. Studies showed that digital intervention is helpful in improving phonological skills. Digital intervention programs have the advantage of providing grading acoustically modified speech signals and image or video resources [32]. Hierarchically auditory and visual cueing is crucial in phonological training [45]. Digital interventions are designed to adapt the level of difficulty and cues of the training according to the success of the child on multiple trials, which might be the reason digital interventions are effective.

Word learning ability has also been proven to be improved through phonological skills digital training, while the conclusion is still controversial. Word learning involves processes such as encoding, working memory, lexical access, and mapping of phonological form to meaning [46,47]. Phonological skills, especially phonemic awareness skills, were proven to be the strongest predictor of children’s word reading ability [48]. Children with DLD might experience impaired mapping of phonological form to meaning, given their less sensitivity to the pronunciation of the words and the information within words during the word learning phase [49]. Digital intervention, featuring visual-auditory stimuli and a game format, has the potential to better engage participants’ attention during the intervention. However, Bishop et al [27] found that participants in the digital intervention group were not better improved in word learning, which might be caused by the short intervention period (4 wk). In addition, it is suggested that children with language impairment and poor reading skills need a more comprehensive approach to improve their reading skills that extends beyond an emphasis on phonemic awareness [32].

General language and speech function was the second most concerned topic in digital interventions. Results showed that only half of the children with DLD gained progress after training. Cohen et al [33] indicated that children with DLD had already received intensive specialist therapy and educational support, while digital interventions were not sufficient in and of themselves to confer additional therapeutic benefits. The varied results between individuals, combined with the generally inconsistent patterns of performance on certain games, create uncertainty as to which elements of FFW are producing improvements [50]. A meta-analysis conducted by Strong et al [51] also indicated that there was no significant effect of FFW on any outcome measure in comparison to active or untreated control groups. Therefore, further studies are needed to evaluate the effectiveness of specific training games or formats of digital interventions.

Studies targeting grammar deficits in DLD showed greater accuracy after intervention but no significant improvement on standardized tests of receptive grammar, or no significant differences in treatment gains between computer-based training and noncomputer-based training. In grammar training games, accuracy could be improved by simply rote learning the meaning of the whole sentence, or by memorizing the correct answers [35]. Therefore, even if children with DLD have difficulties in analyzing the sentence structure, they can still behave well in the training. For this reason, when targeting higher language functions such as grammar, more diverse materials, and learning patterns are needed to promote the generalization of functions. Since the number of studies included in this review was limited, further studies with follow-up periods were needed to examine the effectiveness of digital intervention in receptive and expressive grammar.

In studies targeting vocabulary skills, digital intervention was of promising effectiveness. Children with DLD showed significant improvement after approximately 2-3 months of intervention. Vocabulary training might involve cognitive functions and enhance more global skills [52,53]. Yi et al [38] suggested that children’s language development can promote growth hormone secretion in children, stimulate brain development, promote children’s intellectual development, and have a positive role in improving children’s language communication skills. Therefore, it is recommended that children with DLD receive vocabulary training.

Although digital interventions for DLD were proven effective in several studies, there was evidence showing that DLD did not additionally benefit from digital intervention. Several factors might need to be considered when delivering digital treatment for DLD.

The first factor to be considered is the targeting domain of intervention. In the domains discussed above, digital interventions are demonstrated to be practical and effective in improving basic language and speech skills, such as phonological skills and vocabulary skills. Training of these skills requires lots of repetition and hierarchical audiovisual input, which can be easily achieved by digital techniques [32]. Phonological skills and vocabulary skills are also the prerequisites for learning higher language skills such as grammar and reading [54,55]. However, digital interventions targeting higher language functions, such as grammar, which might involve more cognitive functions [56], were required to be more diversified and more interesting.

Expressive language tended to receive less attention in digital interventions compared to receptive language. Only one study included in this review focused on expressive language. Law et al [57] reported that in-person speech and language therapy interventions were effective for expressive phonological and expressive vocabulary difficulties. However, in computer-based training for expressive language skills, in-time feedback is not provided automatically, which might lower children’s motivation to participate. With the development of computerized language analysis techniques, in-time feedback from the digital program might make expressive language evaluation and training more effective [58,59].

People and places to deliver the intervention might affect the effectiveness of digital interventions. In the included studies, digital interventions were delivered in clinical settings, schools, or at home, and people who carried out digital interventions were speech therapists, school teachers, parents, or a combination of two or all three. Digital language training facilitated by trained therapists in clinical settings was shown to be more effective. The reason might be that in home environments, children are used to playing the game on their own and tend to ignore the instruction during intervention [22]. In addition, interventions delivered in school might take away time from their regular classes, which, to some extent, could be detrimental to their language development [27]. Yi et al [38] applied the intervention pattern with therapists deciding on the training topics and sending them to the patient’s account, and parents led the training at home. This kind of cooperation proved to be beneficial for children during COVID-19, or for children living in remote areas.

The third factor is duration and intensity. The duration of the included studies ranges from approximately 4-12 weeks, and intensity ranges from approximately 15-100 minutes per day. The literature regarding dosage was unclear in optimal intensity, frequency, and duration to maximize efficacy. It seems that interventions carried out for 6 weeks or longer are more effective than those lasting less than 6 weeks. Further investigation of the optimal dosage is necessary. Yi et al [38] and Chen and Lin [29] suggested that the number of training sessions was directly proportional to the performance of children with DLD in language training tasks.

However, the optimal duration and intensity of digital intervention that can meet both treatment needs and avoid the negative effects caused by excessive screen time remain to be resolved. Frizelle et al [60] conducted a systematic review and narrative synthesis and suggested that frequent, short sessions (2/3× per wk, approximately 2 min) and less frequent, long sessions (1× per wk, approximately 20 min) have yielded the best outcomes when composite language measures have been used. For the dosage form, explicit instruction was more beneficial compared with implicit instruction [61]. Variability in input, elicited production, and gestural and other visual supports was beneficial to language development [61]. The amount of screen time was cumulatively and negatively linked to the children’s lexical and general language abilities [62,63]. In addition, the early onset of screen exposure had negative effects on language development [64]. It is suggested that no more than 2 hours of screen time per day has minimal negative effects on development [65]. Better-quality screen use such as educational programs and coviewing was associated with stronger child language skills [63].

The usability and practicality of the digital intervention should also be taken into consideration. Digital intervention for children with DLD uses game-based elements, making the intervention engaging for children. Tablets or mobile phones, with touch screens, provided children with multisensory and direct interaction [22]. Personalization and progress tracking are other advantages of digital intervention. The web-based intervention platform used by Yi et al [38] would automatically pop up the training results, reaction times, and training plan suggestions after each training and automatically adjust the training plan. In addition, digital intervention can offer teachers and families a time-efficient and cost-effective alternative to close learning gaps and avoid the implications of growing disparities in skills due to lengthy waiting times [28]. However, digital intervention might be too distracting and restrict the use of language-facilitating techniques and language interaction between the SLP [22].

Intervention fidelity is an important aspect of designing and implementing intervention effectiveness studies [66]. Most studies have poor treatment fidelity in the areas of “training providers,” “delivery,” and “receipt.” There was a lack of training for the intervention providers, such as parents or school staff. The reason may be related to the characteristics of the digital intervention. In part of the studies, the intervention process is already set in the digital intervention program, which reduces the professional requirements for the providers of the intervention. However, if the treatment protocols were not set in advance in the software, and nonmedical professionals are allowed to use the digital intervention program freely, it is difficult to ensure the effectiveness of the digital intervention for DLD.

### Limitations

This study had some limitations. First, the number of studies regarding the effectiveness of digital intervention in children with DLD in each language domain was insufficient, and most of them have no follow-up period, making it difficult to draw a convincing conclusion. In addition, influential factors such as dosage varied among included studies, and due to the heterogeneity of included studies, the validity of the review analysis might be limited. Besides, this review included research on various research designs, different populations, and interventions. The inclusion of studies with different research designs increases the number of studies included, thereby broadening the perspective on this issue. However, at the same time, the risk of bias is also increasing. In addition, the quality of the included studies was varied, and more rigorous studies were needed in the future. Regarding the intervention domain, more studies focused on the effects of digital interventions for receptive language skills training than for expressive language skills training. Therefore, results showing that receptive language skills training is more effective need to be further examined. Regarding the targeting population, further research could examine the efficacy of digital interventions in children with DLD with different predominant language impairments separately to explore the optimal population for digital interventions.

### Conclusions

This systematic review indicates that phonological skills are the most frequently targeted language domain by digital interventions in children with DLD. Given the limited number and the heterogeneity of the studies included, it is still unclear whether digital intervention was effective in improving different language skills in children with DLD. There is less evidence supporting that digital interventions are effective for expressive language skills. Due to the lack of normality of children’s language development and speech analysis technology, digital programs are unable to respond to children’s errors in a timely manner. Addressing these issues is critical to ensure that mobile health technology is effective in expressive language interventions for children with DLD. Further higher-level evidence, such as RCT studies in this area, is also needed to direct future updates to the digital programs. Digital intervention could be complementary to regular in-person language interventions.

## Supplementary material

10.2196/59992 Multimedia Appendix 1 Description and main outcomes of digital intervention studies for children with developmental language disorder.

10.2196/59992 Multimedia Appendix 2 Fidelity scores of the included studies.

10.2196/59992 Checklist 1 PRISMA (Preferred Reporting Items for Systematic Reviews and Meta-Analyses) checklist.

## Abbreviations

DLD: developmental language disorder
FFW: Fast ForWord
JBI: Joanna Briggs Institute
PRISMA: Preferred Reporting Items for Systematic Reviews and Meta-Analyses
RCT: randomized control trial
SLP: speech and language pathologist
