# Supplementary material for: Digital Intervention in Children With Developmental Language Disorder: Systematic Review
Source: JMIR Mhealth Uhealth. 2025 May 23;13:e59992. doi: 10.2196/59992 (PMC12124036; doi:10.2196/59992)
Supplement: Multimedia Appendix 1 [file mhealth-v13-e59992-s001.docx]

| Speech and language abilities trained | Studies | Gender (M, %) and mean age | No. of participants (Cases and controls) | Intervention | Setting | Provider | Results | Follow-up period | Follow-up result | JBI score (quality) | Study design |
| --- | --- | --- | --- | --- | --- | --- | --- | --- | --- | --- | --- |
| Phonological skills | Bishop et al [27] | Group M: mean age 10.78 ± 1.86;  Group S: mean age 11.19 ± 1.12;  Group U: mean age 10.28 ± 0.88.  Gender of each group was not reported. | N = 36. Modified speech (M, N = 14), ordinary speech (S, N = 13), and untrained control (U, N = 9). | Modified speech (M): FFW, 5 sessions per week, each 15 minutes, 4 weeks; Ordinary speech (S): usual special educational input; Untrained control (U): no intervention. | school | school staff | Children presented with FFW do less well than those trained with ordinary speech. Trained groups did not differ from the untrained group in terms of gains made on standardized tests of spelling or word and nonword reading. | - | - | 9/9 (high) | Quasi-experimental |
| Phonological skills | Carson [28] | M 13 (54.17%), mean age 4.75.  Age and gender of each group were not reported. | N = 24. Intervention (N = 14); Control (N = 10) | Intervention: Reading Doctor (RD) iPad applications, twice a week, each 20-25min, 8-week; Control: teacher-delivered small-group activities related to letters and sounds. | preschool | teachers, SLP students and initial teacher education (ITE) students. | Children in the intervention group performed significantly better than children in the control condition in phoneme blending, phoneme segmentation, letter-sound recognition, accuracy of phoneme–grapheme conversions. | - | - | 7/13 (moderate) | RCT |
| Phonological skills | Chen and Lin [29] | Intervention: mean age 5.85 ± 0.53; Control: mean age 5.65 ± 0.53.  Gender of each group was not reported. | N = 49. Intervention (N = 34); Control (N = 15). | Intervention: 作業TipOn, 1 session per week, each 1 hour, 9 weeks; Control: delayed intervention | home | parents | Children in the experimental group exhibited significantly greater pre-post gains in word definition tasks and lexical tone than the control group. Positive relationship between the total number of phonetic games played and the pre-post gain in the word definition production task. | - | - | 8/13 (moderate) | RCT |
| Phonological skills | Dacewicz et al [30] | Experiment group: M 13 (72.22%), mean age 6.3 ± 1.0;  Control group: M 13 (72.22%), mean age 6.0 ± 0.8. | N = 36. Experiment group (N = 18); Control group (N = 18). | Experiment: Dr. Neuronowski, four sessions per week, each 1 hour, 6 weeks; Control: other computer games for identification and discrimination of syllables and words, four sessions per week, each 1 hour, 6 weeks. | a separate room at the Nencki Institute or the Early Intervention Centre. | a trained consultant | In the experimental group, MMN amplitude enhancement was observed in both ISI conditions. In both experimental and control groups, P3a amplitude was enhanced in both ISIs. | - | - | 9/13 (moderate) | RCT |
| Phonological skills | Heikkilä et al [31] | Audiovisual training group: M 6 (60.00%), mean age 8.75;  Auditory training group: M 7 (70.00%), mean age 9.08. | N = 20. Audiovisual training group (N = 10); Auditory training group (N = 10). | Audiovisual: program developed by authors and run with Presentation software, 5 days a week, each 10-15 minutes, 6 weeks; Auditory: same program with the screen blocked. 5 days a week, each 10-15 minutes, 6 weeks. | Valteri Onerva School | SLP | Training with audiovisual speech can improve the phonological skills of children with DLD in the repetition of nonsense words. Audiovisual speech might be more effective than auditory speech in training phonological skills in children. | - | - | 9/9 (high) | Quasi-experimental |
| General | Loeb et al [32] | FFW: M 14 (58.33%), mean age 7.42 ± 9.97;  CALI: M 17 (58.62%), mean age 7.33 ± 11.35;  ILI: M 17 (68.00%), mean age 7.58 ± 8.72;  AC: M 18 (72.00%), mean age 7.33 ± 10.48. | N = 103. FFW (N = 24), CALI (N = 29), ILI (N = 25), AC (N = 25). | FFW: FFW, 5 days a week, each 100 min, 6 weeks; CALI: other software targeted phonological skills, 5 days a week, each 100 min, 6 weeks; ILI: literature-based intervention, 5 days a week, each 100 min, 6 weeks; AC: software not for language development, 5 days a week, each 100 min, 6 weeks. | School | SLP | Children in the FFW, CALI, and ILI conditions showed significant improvement in blending sounds compared to the AC group immediately after the test. | 6 mo | After 6 mo, long-term gains were moderate for blending sounds, but not significant. | 8/9 (high) | Quasi-experimental |
| General | Cohen et al [33] | FFW: M 16 (69.57%), mean age 7.34 ± 1.29;  Computer software: M 22 (81.48%), mean age 7.43 ± 1.21;  Control: M 17 (62.96%), mean age 7.41 ± 1.17. | N = 77, FFW (N = 23); Computer software (N = 27); Control (N = 27). | Group A: FFW, 5 days a week, each 90 min, 6 weeks; Group B: Computer software, educational software packages, 5 days a week, each 90 min, 6 weeks; Group C: Control. No intervention. | home | parents | Each group made significant gains in language scores, but there was no additional effect for either computer intervention. | 6 mo | Group A showed significant gains in the scores of Expressive and Receptive Language on the CELF–3UK and the rhyming skills. | 9/13 (moderate) | RCT |
| General | Gillam et al [34] | CALI: M 34 (62.96%), mean age 7.42;  FFW: M 29 (53.70%), mean age 7.50;  ILI: M 38 (70.37%), mean age 7.67;  AE: M 35 (64.81%), mean age 7.58. | N = 216. CALI (N = 54), FFW (N = 54), ILI (N = 54), AE (N = 54). | CALI (computer-assisted language intervention): software for phonological skills, 5 days a week, each 100 min, 6 weeks; FFW: FFW, 5 days a week, each 100 min, 6 weeks; ILI (individualized language intervention): literature-based, 5 days a week, each 100 min, 6 weeks; AE (academic enrichment): computer software not for language development, 5 days a week, each 100 min, 6 weeks. | a quite area | SLP | FFW was not more effective at improving general language skills or temporal processing skills than a nonspecific comparison treatment (AE) or specific language intervention comparison treatments (CALI and ILI) that did not contain modified speech stimuli. | 3 mo and 6 mo | Participants in the FFW-L and CALI conditions earned higher phonological awareness scores than children in the ILI and AE conditions at the 6-month follow-up testing. | 12/13 (high) | RCT |
| Grammar-receptive | Hsu and Bishop [35] | DLD-trained: mean age 8.6 ± 1.32;  TD-Grammar-matched: mean age 9.1 ± 1.32;  TD-age-matched: mean age 5.8 ± 0.86;  DLD-untrained: mean age 8.9 ± 0.77. Gender of each group was not reported. | N = 96. DLD-trained (N = 28); TD-Grammar-matched (N = 28); TD-age-matched (N = 20); DLD-untrained (N = 20). | DLD-trained: FFW, 5-7 min per day, 4 sessions in 4-6 days; TD-Grammar-matched: FFW, 5-7 min per day, 4 sessions in 4-6 days; TD-age-matched: FFW, 5-7 min per day, 4 sessions in 4-6 days; DLD-untrained: no intervention | not mentioned | not mentioned | Children with DLD showed greater accuracy with repeated sentences compared with unique sentences. Training did not improve children’s performance on a standardized test of receptive grammar. | - | - | 7/13 (moderate) | RCT |
| Grammar-receptive | Bishop et al [36] | Group M: mean age 11.08 ± 1.13;  Group S: mean age 10.85 ± 1.79;  Group U: mean age 10.28 ± 0.88.  Gender of each group was not reported. | N = 36. Modified speech (M, N = 13), ordinary speech (S, N = 15) and untrained control (U, N = 9). | Modified speech (M): FFW, 5 sessions per week, each 15 minutes, 4 weeks; Slow speech (S): trainings with a 1.2-second delay between the end of one sentence segment and the start of the next; Untrained group (U): continued to receive their regular educational input. | school | school staff | Responses speeded up, and most children performed well above chance, accuracy typically remained below 95% correct. Trained groups did not differ from untrained children on language or auditory outcomes. Acoustically modified speech input did not enhance comprehension. | - | - | 9/9 (high) | Quasi-experimental |
| Grammar-expressive | Washington et al [37] | CAT: M 8 (72.73%), age range 3.92 ~ 4.50;  nCAT: M 8 (72.73%), age range 4.17 ~ 4.83;  No treatment: M 11 (91.67%), age range 3.50 ~ 4.92. | N = 34. CAT (N = 11); nCAT (N = 11); No treatment (N = 12). | CAT (computer-assisted treatment): My sentence builder, once a week, each 20 min, 10 weeks; nCAT (non-computer-assisted treatment): ‘‘table-top’’ procedures, once a week, each 20 min, 10 weeks; No treatment: children awaiting treatment. | not mentioned | SLP | C-AT and nC-AT participants significantly outperformed controls pre-to-post to 3 mo post-treatment in both assessment contexts. No significant differences in treatment gains were found between C-AT and nC-AT. | 3 mo | C-AT and nC-AT participants significantly outperformed controls pre-to-post to 3 mo post-treatment in both assessment contexts. | 8/13 (moderate) | RCT |
| Vocabulary-receptive | Yi et al [38] | Intervention: M 44 (52.38%), mean age 4.88 ± 0.79;  Control: M 42 (53.85%), mean age 4.69 ± 0.85. | N = 162. Intervention (N = 84); Control (N = 78). | Intervention: Jingyun Rehab Platform, daily task, 3 mo; Control: conventional home-based rehabilitation recommendations, 3 mo. | home | parents | Children with DLD in the cloud-based rehabilitation group performed significantly better in language abilities than the control group. The frequency of training sessions was proportional to their performance on language, memory, and cognition tasks. | - | - | 9/9 (high) | Quasi-experimental |
| Vocabulary-receptive | Zwitserlood et al [22] | Intervention: M 26 (72.22%), mean age 3.47 ± 0.17;  Control: M 25(69.44%), mean age 3.53 ± 0.21. | N = 72. Intervention (N = 36); Control (N = 36). | Intervention: My PlayHome, 12 sessions in an 8-9-week period, each 10-min; Control: intervention based on real objects, 12 sessions in an 8–9-week period, each 10-min. | daycare center | SLP | Children in both groups learned significantly more target words than control words. No significant differences in gains between the two intervention conditions were found. | 5 weeks | At the end of the 5-week retention period, there was a significant growth in target words, and the newly learned words were still known. | 8/13 (moderate) | RCT |
